# Selective Data Analysis in Brown et al.'s Continued Critical Reanalysis

**DOI:** 10.1371/journal.pone.0160565

**Published:** 2016-08-04

**Authors:** Barbara L. Fredrickson

**Affiliations:** Department of Psychology and Neuroscience, University of North Carolina at Chapel Hill, Chapel Hill, North Carolina, United States of America; University of Illinois-Chicago, UNITED STATES

In their latest critique [1], Brown et al. verify the primary statistical results of our 2015 PLoS ONE report [2]. The results Brown et al. report for their mixed effect linear model analyses of our Confirmation study and pooled Discovery and Confirmation studies in their Table 3 [1] are nearly identical to the results we reported in our Tables 2 and 3 [2].

Nevertheless, Brown et al. continue to dispute the conclusions that follow from these results. They do so by selectively re-analyzing our Discovery study dataset (*N* = 76), which represents only 25% of the data presented in our 2015 report. Using this approach, Brown et al. argue that the relationship between eudaimonic well-being and gene expression is sensitive to (1) the inclusion vs. exclusion of a single data case (SOBC1-1293), and (2) the effects of a coding error in the originally posted covariate data for another data case (SOBC1-1299). However, analysis of the full set of data presented in our Discovery and Confirmation studies (*N* = 198) reveals that the association of eudaimonic well-being with gene expression is not materially affected by either of these factors (see Table 1 herein).

**Table 1:** Association of well-being with gene expression: pooled Discovery and Confirmation Studies (omitting Discovery study participant SOBC1_1293 or using uncorrected race covariate value for Discovery study participant SOBC1_1299).

	Well-being dimension	Association *b* ± SE^1^	Test Statistic	*p*-value
**A. 2-dimensional**				
Primary analyses^2^	Hedonic	0.074 ± 0.042	*t*(179) = 1.77	.0781
Omitting SOBC1_1293	Hedonic	0.047 ± 0.041	*t*(178) = 1.15	.2517
Uncorrected SOBC1_1299	Hedonic	0.079 ± 0.042	*t*(179) = 1.88	.0619
Primary analyses^2^	Eudaimonic	-0.116 ± 0.043	*t*(179) = -2.71	.0074*
Omitting SOBC1_1293	Eudaimonic	-0.102 ± 0.042	*t*(178) = -2.42	.0165*
Uncorrected SOBC1_1299	Eudaimonic	-0.115 ± 0.043	*t*(179) = -2.69	.0077*
**B. 3-dimensional**				
Primary analyses^2^	Hedonic	0.059 ± 0.042	*t*(178) = 1.39	.1663
Omitting SOBC1_1293	Hedonic	0.037 ± 0.042	*t*(177) = 0.88	.3775
Uncorrected SOBC1_1299	Hedonic	0.063 ± 0.042	*t*(178) = 1.49	.1372
Primary analyses^2^	Psychological	0.015 ± 0.052	*t*(178) = 0.29	.7702
Omitting SOBC1_1293	Psychological	0.003 ± 0.052	*t*(177) = 0.05	.9586
Uncorrected SOBC1_1299	Psychological	0.016 ± 0.052	*t*(178) = 0.32	.7522
Primary analyses^2^	Social	-0.126 ± 0.045	*t*(178) = -2.81	.0055*
Omitting SOBC1_1293	Social	-0.103 ± 0.045	*t*(177) = -2.31	.0220*
Uncorrected SOBC1_1299	Social	-0.127 ± 0.045	*t*(178) = -2.82	.0053*
Primary analyses^2^	Eudaimonic (PWB & SWB)^3^	-	*F*(2,178) = 5.25	.0061*
Omitting SOBC1_1293	Eudaimonic (PWB & SWB)^3^	-	*F*(2,177) = 3.89	.0223*
Uncorrected SOBC1_1299	Eudaimonic (PWB & SWB)^3^	-	*F*(2,178) = 5.24	.0061*
**C. Alternative 3-dimensional**^**4**^				
Primary analyses^2^	Hedonic	0.032 ± 0.043	*t*(178) = 0.74	.4589
Omitting SOBC1_1293	Hedonic	0.019 ± 0.043	*t*(177) = 0.43	.6666
Uncorrected SOBC1_1299	Hedonic	0.037 ± 0.043	*t*(178) = 0.85	.3991
Primary analyses^2^	Psychological	0.032 ± 0.049	*t*(178) = 0.65	.5173
Omitting SOBC1_1293	Psychological	0.012 ± 0.049	*t*(177) = 0.23	.8149
Uncorrected SOBC1_1299	Psychological	0.035 ± 0.049	*t*(178) = 0.71	.4809
Primary analyses^2^	Social	-0.144 ± 0.035	*t*(178) = -4.17	< .0001*
Omitting SOBC1_1293	Social	-0.116 ± 0.035	*t*(177) = -3.32	.0011*
Uncorrected SOBC1_1299	Social	-0.146 ± 0.035	*t*(178) = -4.22	< .0001*
Primary analyses^2^	Eudaimonic (PWB & SWB)^3^	-	*F*(2,178) = 9.52	.0001*
Omitting SOBC1_1293	Eudaimonic (PWB & SWB)^3^	-	*F*(2,177) = 6.46	.0020*
Uncorrected SOBC1_1299	Eudaimonic (PWB & SWB)^3^	-	*F*(2,178) = 9.69	.0001*

^1^ Partial regression coefficients relating standardized gene expression values to standardized scores on 2-d and 3-d representations of well-being (A, B, C). All associations are adjusted for age, sex, race, BMI, smoking, alcohol consumption, illness symptoms, and gene transcript covariates marking major leukocyte subsets.

^2^ Primary analyses were reported in [2].

^3^ 3-d representations of overall well-being involve a 2-d representation of eudaimonic well-being (i.e., distinct subdomains of Social Well-Being [SWB] and Psychological Well-Being [PWB]). The aggregate association of 2-d eudaimonic well-being with gene expression is tested by an omnibus *F* ratio comprising the 2 dimension-specific partial regression coefficients listed above.

^4^ The alternative 3-d representation derives from Brown et al.’s factor analyses reallocating 2 questionnaire items from the social wellbeing measure to the measure of psychological well-being [1, 5].

* *p*-values < .05 are highlighted to facilitate comparison of significance across alternative analyses.

The mixed effect linear model analyses reported in Table 1 account for correlation among the multiple indicator genes examined [3] and continue to indicate a significant inverse relationship between eudaimonic well-being and gene expression, regardless of SOBC1-1293 exclusion or the SOBC1-1299 coding error. (Because SOBC1-1293 and SOBC1-1299 come from the Discovery study sample, they have no effect on analyses of the Confirmation study dataset alone [*N* = 122] or the Generalization study dataset [*N* = 107].) The Discovery study sample alone is too small to provide a well-powered mixed effect linear model analysis. Thus, it is unsurprising that Brown et al.’s Table 4 [1] shows non-significant regression coefficients for eudaimonic well-being and point estimates that vary substantially from those of the better-powered analyses of the Confirmation study and the pooled Discovery and Confirmation studies (reported in our Tables 2 and 3, respectively [2], and Brown et al.’s Table 3 [1]). This discrepancy in statistical power between Brown et al.’s selective reanalyses (reported in their Table 4) and a more complete analysis (replicated in their Table 3) is evident in the larger Standard Errors (SE) in their Table 4 versus Table 3 [1].

In their previous critique of our 2013 report [4] on gene expression correlates of well-being, Brown et al. [5] argued for the replication of findings in additional samples using mixed effect linear model analyses. Such data are now available from two new samples with 229 new participants, and results continue to indicate a significant inverse relationship between eudaimonic well-being and gene expression. Brown et al.’s claims of statistical instability rely on selective omission of these new data, which comprise 75% of the data presented in our 2015 PLoS ONE report.
